# Au Nanoparticle Sub-Monolayers Sandwiched between Sol-Gel Oxide Thin Films

**DOI:** 10.3390/ma11030423

**Published:** 2018-03-14

**Authors:** Enrico Della Gaspera, Enrico Menin, Gianluigi Maggioni, Cinzia Sada, Alessandro Martucci

**Affiliations:** 1 School of Science, RMIT University, Melbourne 3000, Australia; enrico.dellagaspera@rmit.edu.au; 2 Department of Industrial Engineering, University of Padova, via Marzolo 9, Padova 35131, Italy; enrico.menin@unipd.it; 3 Materials and Detectors Division, INFN, Legnaro National Laboratories, Viale dell’Università, Legnaro 35020, Italy; Gianluigi.Maggioni@lnl.infn.it; 4 Department of Physiscs and Astronomy, University of Padova, via Marzolo 8, Padova 35131, Italy; cinzia.sada@unipd.it; 5 National Research Council of Italy, Institute for Photonics and Nanotechnologies, Padova, via Trasea 7, Padova 35131, Italy

**Keywords:** metal oxides, multi-layer, surface plasmon resonance, optical sensors

## Abstract

Sub-monolayers of monodisperse Au colloids with different surface coverage have been embedded in between two different metal oxide thin films, combining sol-gel depositions and proper substrates functionalization processes. The synthetized films were TiO_2_, ZnO, and NiO. X-ray diffraction shows the crystallinity of all the oxides and verifies the nominal surface coverage of Au colloids. The surface plasmon resonance (SPR) of the metal nanoparticles is affected by both bottom and top oxides: in fact, the SPR peak of Au that is sandwiched between two different oxides is centered between the SPR frequencies of Au sub-monolayers covered with only one oxide, suggesting that Au colloids effectively lay in between the two oxide layers. The desired organization of Au nanoparticles and the morphological structure of the prepared multi-layered structures has been confirmed by Rutherford backscattering spectrometry (RBS), Secondary Ion Mass Spectrometry (SIMS), and Scanning Electron Microscopy (SEM) analyses that show a high quality sandwich structure. The multi-layered structures have been also tested as optical gas sensors.

## 1. Introduction

There is a growing need for nanostructured materials with tailored optical and electrical properties, however the material itself does not always provide the required properties: for this reason, a combination of different materials with accurately controlled organization is sometimes necessary in order to enhance the device performances and/or to acquire new properties. In this regard, the combination of semiconducting oxides and noble metals has been extensively investigated for applications in several fields, including photocatalysis, sensing, optoelectronics, and energy conversion [1,2,3]. The presence of noble metals on the surface of metal oxides enables efficient charge separation and electron transfer in optoelectronics devices, but also enhanced optical properties if the noble metals show localized Surface Plasmon Resonance (SPR) peaks in the spectral range of interest. This is usually the case for gold and silver, which have found use in many oxide-based nanocomposites, for example, for enhanced photocatalysis and solar fuel generation [4,5].

The discovery of the strong SPR coupling of close packed Au and Ag nanoparticles (NPs) [6], which leads to an increase of the intensity of the local electromagnetic field in the immediate surroundings of the metal particles, has driven an additional research effort that is devoted to the precise assembly of plasmonic NPs and their integration within optoelectronic devices. Several reports have been published discussing the distinctive optical and electrical properties of two-dimensional arrays of Au NPs, which can be exploited for different applications for example in Surface Enhanced Raman Scattering (SERS), sensing, and catalysis [7,8,9,10,11]. The combination of these ordered assemblies of Au NPs with catalytically and/or electrically active materials, such as semiconducting metal oxides, can generate a synergistic effect between the two components, enhancing the overall nanocomposite properties, for example, in optical recognition of reducing gases and Volatile Organic Compounds (VOCs) [9,12]. This nano-engineering of precisely ordered metal nanostructures and oxide surfaces can be achieved with a variety of experimental techniques, including lithography, sputtering, Chemical Vapor Deposition (CVD), and ion implantation. However, all of these techniques require either complex synthetic procedures or expensive equipment, and sometimes both. In this work, we present a simple and straightforward approach to synthesize high quality oxide/metal nanocomposites where plasmonic nanoparticles are assembled in a close-packed fashion, and interfaced two different metal oxides. We expand on our previous study on Au colloids deposited on properly functionalized substrates, and then covered with metal oxides [10], and by using only wet-chemistry techniques, we fabricate sub-monolayer of Au NPs that are sandwiched between two metal oxide layers. In detail, layers of monodisperse Au NPs are deposited over a semiconductive sol-gel film (NiO, TiO_2_) and are then covered with a different sol-gel layer (TiO_2_, NiO, ZnO). Within these structures, the Au NPs layer faces one material on one side and a different material on the other side, with potentially exciting electrical and optical properties that can find applications in several fields, including optoelectronic devices [13], sensors [14], and photovoltaics [15]. In addition to the simplicity of the presented method, such a synthetic procedure can be easily extended for many other metal oxides coatings, and to more complex multi-layered structures with different metal NPs that are embedded in between different semiconducting layers.

## 2. Materials and Methods

Spherical Au NPs of about 13 nm in diameter were synthesized with the Turkevich method by reducing Au ions in water at 100 °C with sodium citrate. The whole synthetic and purification protocol has been described previously [10].

To deposit a TiO_2_ layer, a solution of Ethanol (0.413 mL), titanium butoxide (0.447 mL), and acetylacetone (0.216 mL) was prepared under vigorous stirring at room temperature. After 10 min, 0.1 mL Milli-Q water were added and were let stir for additional 20 min. Then, 1.83 mL ethanol was added, the total solution was let stir for 5 more minutes, and then it was used for films deposition.

To deposit the NiO layer, 300 mg of Nickel Acetate tetrahydrate were dissolved in 2 mL methanol, and subsequently 0.18 mL diethanolamine were added under stirring. After 40 min, 1.4 mL ethanol were added, and after additional 5 min, the solution was used for films deposition.

To deposit the ZnO layer, 200 mg of Zinc acetate dehydrate were dissolved in 0.9 mL ethanol, and subsequently 0.066 mL monoethanolamine (MEA) were added under stirring. After 30 min, 0.35 mL ethanol are added and the solution was used for film depositions after five more minutes of stirring.

The bottom oxide coating was deposited on either Si or SiO_2_ (fused silica) substrates by spin coating with rotating speed ranging from 2000 rpm to 3000 rpm for 30 s, and then the sample was annealed directly at 500 °C for 10 min. The spinning rate was calibrated and adjusted in order to obtain films of about 45 nm after the 500 °C annealing for all of the three oxides used. The accuracy and reproducibility of the spinning procedure was tested after repeated depositions and gave a ±5 nm error on the sample thickness. To promote Au NPs bonding, the outer oxide surface is functionalized with aminopropyltrimethoxsilane (APS), after re-activation of the surface to promote formation of hydroxyl groups, which are necessary for the reaction with APS molecules (as a consequence of the thermal annealing, all of the hydroxyl groups were removed). The optimized activating procedure for NiO and TiO_2_ films consisted in dipping the samples into a 4% H_2_O_2_ aqueous solution at room temperature for 1 min, followed by a thorough rinsing with deionized water. After this procedure, the previously reported protocols of substrate functionalization and Au NPs layer deposition were performed [10], followed by the deposition of the top oxide layer using the sol-gel recipes described earlier; eventually, the samples were thermally treated at 500 °C for one hour. The surface coverage of Au NPs was tailored simply diluting the Au colloidal solution: in this study we prepared samples with three different Au surface coverages, hereafter indicated as low (L, 6%), medium (M, 19%), and high (H, 35%). The surface coverage was estimated from SEM images, as reported in reference [10]. ZnO was not used as bottom layer because the hydroxylation protocol caused etching of the porous ZnO films, even if it was performed in milder conditions.

The films were characterized by XRD using a Philips PW1710 diffractometer (Amsterdam, The Netherlands) equipped with glancing-incidence X-ray optics. The analysis was performed at 0.5° incidence, using CuKα Ni filtered radiation at 30 kV and 40 mA. Optical absorption spectra of samples that were deposited on fused silica substrates were measured in the 300–2000 nm range using a Jasco V-570 spectrophotometer (Japan) Ellipsometry measurements were carried out on a J.A. Woollam V-VASE Spectroscopic Ellipsometer (Lincoln, NE, USA) in vertical configuration, in the 300–1500 nm range at three different angles of incidence (65°, 70°, 75°). The nanocomposites were modeled with Cauchy dispersions for the non-absorbing region, while Gaussian or Tauc-Lorentz oscillators were used for the UV absorption onset fitting. Rutherford backscattering spectrometry (RBS) was performed with an electrostatic accelerator, Van de Graaff type, using single-charged alpha particles (^4^He^+^) at 2.0 MeV and 20 nA. RBS analysis was performed on samples deposited on Si substrates. The incident beam was perpendicular to the sample, while the scattering angle was 160°. The surface and cross-sectional structure of the nanocomposite films were investigated with a xT Nova NanoLab Scanning Electron Microscopy (SEM). Secondary Ion Mass Spectrometry (SIMS) was exploited to measure the elemental in-depth profiles of chemical species in the deposited film. SIMS measurements were carried out by means of an IMS 4f mass spectrometer (Cameca, Padova, Italy), using a 14.5 KeV Cs^+^ primary beam and by negative secondary ion detection. The charge build up while profiling the insulating samples was compensated by an electron gun without any need to cover the surface with a metal film. The SIMS spectra were carried out at different primary beam intensity (20 nA, stability 0.2%) rastering over a 150 × 150 µm^2^ area and detecting secondary ions from a sub region close to 7 × 7 µm^2^ to avoid crater effects. The primary beam was chosen in order to optimise the depth resolution and the multilayer interface determination. The signals were detected in beam blanking mode (i.e., interrupting the sputtering process during magnet stabilization periods) in order to improve the in-depth resolution. Moreover, the dependence of the erosion speed on the matrix composition was taken into account by measuring the erosion speed at various depths for each sample. The erosion speed was then evaluated by measuring the depth of the erosion crater at the end of each analysis by means of a Tencor Alpha Step profilometer with a maximum uncertainty of few nanometers (final value given by the average on 8 measures). The measurements were performed in High Mass Resolution configuration to avoid mass interference artifacts. The film thickness was determined by analysing the element signal dynamics. The error of the film thickness contains, therefore, contributes of the element inter-diffusion, of the film roughness, and finally of the technique artefacts. Optical gas sensing tests were performed by making optical absorption measurements in the 350–1500 nm wavelength range on films deposited on SiO_2_ glass substrates using a Harrick gas flow cell (with an optical path length of 5.5 cm), coupled with a Jasco V-570 spectrophotometer. The operating temperature (OT) was set at 300 °C and gases at concentrations of 1 vol% for H_2_ and of 1 vol% for CO in dry air at a flow rate of 0.4 L/min were used. The incident spectrophotometer beam was set normal to the film surface and illuminated an area of ~13 mm^2^.

## 3. Results and Discussion

As described in the experimental section, the different samples that were prepared consist on a bottom layer (TiO_2_, NiO), an intermediate layer of Au NPs with different surface coverage, and a top layer (TiO_2_, NiO, ZnO). The Au colloids surface coverage can be easily tuned by changing the NPs concentration in the spin coating solution (more concentrated solutions lead to greater surface coverages) or by modifying the spinning speed (increasing the rotational speed leads to lower surface coverages).

**Figure 1:**
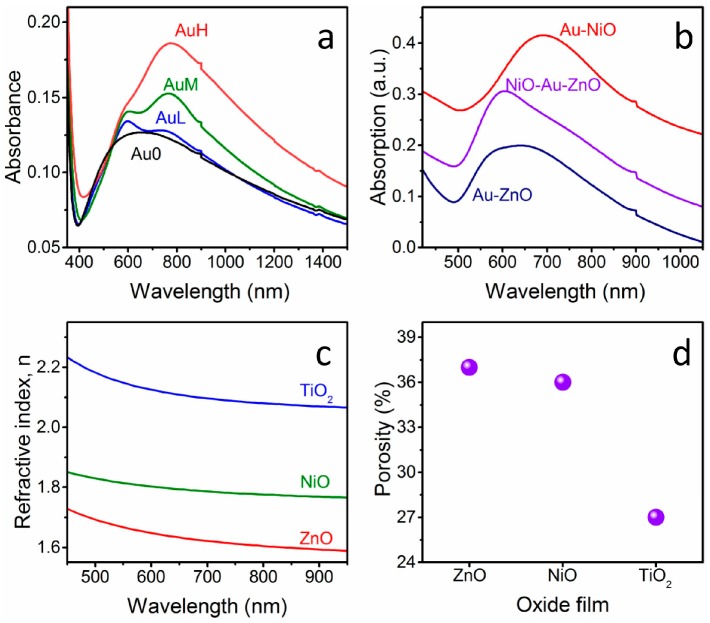
(**a**) Optical absorption spectra of TiO_2_-NiO double layer with different Au nanoparticles (NPs) amount embedded in between. Au surface coverages: L = 6%, M = 19% and H = 35%; (**b**) Optical absorption spectra of Au-ZnO, Au-NiO and NiO-Au-ZnO nanocomposites deposited on fused silica susbtrate; (**c**) Refractive index dispersion curves for the three oxide films after the 500 °C annealing; and, (**d**) Porosity of the three oxide films evaluated with the Bruggeman model from the ellipsometric measurements.

Optical spectroscopy is a powerful tool to investigate Au NPs amount and organization in between the two oxide layers. Figure 1 shows the absorption spectra of some of the prepared nanostructures: for all of the NPs-containing samples the SPR peak appears in the visible-near IR range (Figure 1a,b). As can be seen in Figure 1a, an increase in intensity of the SPR peak component at higher wavelengths with increasing Au NPs surface coverage is observed, as already reported previously for bare Au NPs layers [8,9], and for Au NPs layers that were covered with metal oxides [10]. This effect is due to the reduced mutual distance between close-packed Au NPs, which leads to a stronger coupling of the plasmon resonances [6]. The broad absorption feature of the Au-free sandwich structure is due to optical interference because of the high refractive index of the oxide films (see below).

By comparing a sandwich structure that was prepared in this study with Au NPs layers that were deposited on glass with the same particles density and covered with only one metal oxide (Figure 1b), it can be noticed that the optical features of Au NPs that are embedded in between two metal oxides (in this case NiO as bottom layer and ZnO as top layer) are effectively in between the properties of the Au-NiO and Au-ZnO systems: in fact, the SPR peak of Au NPs deposited on glass and covered with ZnO is registered at about 605 nm, while when the Au colloids deposited on glass are covered with NiO, the SPR frequency is 690 nm. The plasmon peak of the Au NPs embedded in between the two oxides is definitely blue shifted compared to Au-NiO films, while due to the low frequency component related to plasmon coupling of neighboring NPs, it is difficult to appreciate the red shift compared to Au-ZnO. Nevertheless, the low frequency component of the SPR band is definitely red shifted in the sandwich structure compared to the Au-ZnO composite. 

The blue or red shift observed in the SPR band is related to the difference in refractive index between the two oxides: NiO has a higher refractive index value when compared to ZnO, as will be discussed later on along with the ellipsometry measurements. So, according to Mie theory [16], the greater the refractive index value of the matrix in which the Au NPs are embedded, the greater the SPR wavelength arising from the metal NPs.

The actual refractive index n and the thickness of the oxide layers that were deposited on glass substrates have been measured using spectroscopic ellipsometry, and the results are presented in Figure 1c. As can be seen, the refractive index values for the three oxides that are used are rather different between each other, but also they differ from the bulk values for the respective oxides. This difference is ascribed to the residual porosity of the thin films, which is a well-known effect for oxides films that are prepared from sol-gel solutions and annealed at relatively low temperatures, outside the sintering range [17,18,19]. For this reason, the oxide layer is modeled as an effective medium that is composed of dense matrix and pores, and through effective medium approximation (EMA) models, it is possible to evaluate the porosity amount. Using the bulk refractive index values at ~600 nm for the three oxides (n_ZnO_ = 2.01 [20]; n_NiO_ = 2.33 [21]; n_TiO2_ = 2.51 [20]), the pores volume fraction evaluated with the Bruggeman [22] relationship are 37%, 36%, and 27% for ZnO, NiO, and TiO_2_, respectively. As a consequence, as can be also visualized in Figure 1c,d, the actual refractive index of the prepared samples follows the order of the bulk and dense materials, but the porosity of the TiO_2_ layer is lower when compared to the other two oxides. In fact, anatase layers are more compact and smooth as compared to NiO and ZnO, as will be clarified later along with SEM characterization.

**Figure 2:**
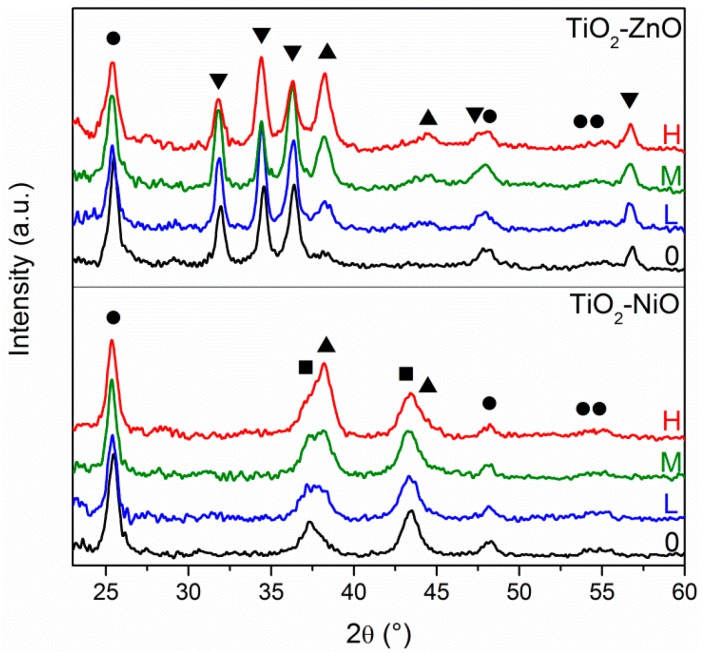
XRD patterns of TiO_2_-NiO and TiO_2_-ZnO series; Au NPs surface coverage in the different samples is highlighted with H (high), M (medium), L (low) and 0 (zero). Predicted diffraction peaks positions for TiO_2_ (●), NiO (■), ZnO (▼) and Au (▲) according to the respective ICDD cards are also reported.

X-ray diffraction analysis gives a confirmation of the different Au amount according to the concentration of the solutions that are used for the Au layer deposition, and also verifies the crystallinity of the three oxides: all of these results are reported in Figure 2. Typical diffraction patterns for anatase TiO_2_ (ICDD No. 86-1157, highlighted with ●), bunsenite NiO (ICDD No. 47-1049, highlighted with ■), wurtzite ZnO (ICDD No. 36-1451, highlighted with ▼) and cubic Au (ICDD No.04-0784, highlighted with ▲) can be easily identified in the prepared samples, according to their respective composition. Analyzing the oxide diffraction peaks, they do not undergo any relevant change from one sample to another, nor in the intensity or broadening (the full width at half maximum, FWHM, is related to the crystallite size, according to the Scherrer equation), validating the reproducibility of the different sol-gel recipes adopted. However, it has to be said that such a comparison, especially for the intensity of the diffraction peaks, is merely qualitative. In fact, although all of the samples had approximately the same films thickness and the same substrate size, the difference in XRD peaks intensity is strongly related to the thickness of the samples, the X-ray beam spot size, the careful alignment of the sample stage, because the measurements have been performed at glancing angle (0.5°), and so a quantitative comparison would be rather speculative. As far as Au diffraction peaks are concerned, few differences can be observed among the different samples: by increasing the Au NPs amount (from Low, to Medium, to High), a clear progressive increase in Au peaks intensity is detected, confirming the different surface coverage.

XRD has also been adopted to evaluate the effect of the activation of the bottom oxide layer before performing the APS functionalization process: as described in the experimental section, NiO and TiO_2_ films were immersed in a hydrogen peroxide dilute solution in order to create –OH surface bonds. XRD measurements performed before and after the etching treatment (not reported) do not show any modification of the oxide diffraction peaks, nor in intensity or FWHM, excluding any change in the morphology and chemical composition of the oxide layers. 

**Figure 3:**
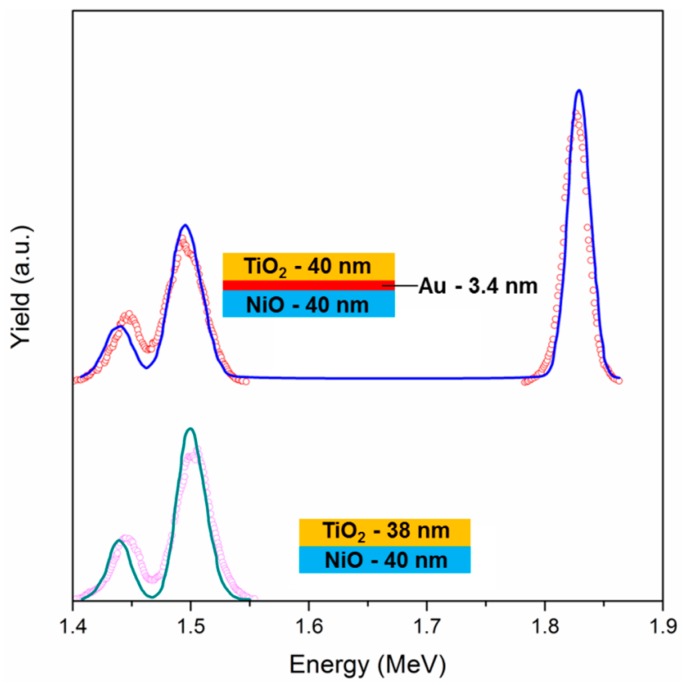
RBS spectra (open circles) and respective simulations (straight lines) of NiO-TiO_2_ sample with and without an interlayer of Au NPs. The schematic representation of the layer stacks used for the fitting is also shown.

NiO-TiO_2_ sandwich structures—with NiO as bottom layer and TiO_2_ as top layer—with and without Au NPs, have been characterized with Rutherford Backscattering Spectrometry (RBS): This technique is useful to gain information about thickness, composition, and spatial distribution over thickness of the different components. The spectrum of the Au-free sample (Figure 3) shows two distinct peaks, which are centered at about 1.44 MeV and 1.5 MeV, which can be ascribed to Ti and Ni signals, respectively. The predicted energy positions for Ti and Ni (with the experimental setup used) are 1.44 MeV and 1.53 MeV, respectively: Ni signal is found at lower energies because the NiO layer is slightly far from the surface, so it is probed after the TiO_2_ film. A simulation has been performed when considering a simple sandwich structure composed of a bottom layer of NiO and a top layer of TiO_2_, letting the thickness vary: the best fit was obtained with a TiO_2_ layer of 38 nm and a NiO film of 40 nm. The two values are extremely close to each other, confirming the correct choice of the deposition parameters in order to get similar thicknesses, even if the thickness values are slightly lower when compared to the expected ones (about 45 nm), as measured by ellipsometry and SEM analyses (see below): this is because both SEM and ellipsometry take into account the porosity of the films, while the RBS technique is based on nominal density for the different materials, and measuring the atoms/cm^2^ values, the apparent thickness evaluated with RBS is reliable only if measuring fully dense materials. Nonetheless, having obtained similar thicknesses for both oxide coatings is a further proof of the accuracy of the experimental procedure.

The same sample architecture, but with a layer of Au NPs in between the two oxides (Figure 3), shows the same two peaks at 1.44 MeV and 1.5 MeV, and an additional peak at 1.82 MeV, due to metallic Au. These experimental data have been modeled using a bottom layer of fully dense NiO of 40 nm thickness, an intermediate layer of Au (3.4 nm) and a top layer of dense TiO_2_ of 40 nm. Again, the two oxide films are of the same thickness, slightly lower than the expectations due to the porosity effect described before. Since the software that is used for the fitting procedure does not take into account the possibility of having a layer composed of NPs, the simulation has been carried out with a bulk gold layer, obtaining a thickness of 3.4 nm as best fit. Using the integral of the Au peak, the dose of Au atoms can be estimated, being it 2 × 10^16^ at/cm^2^; knowing the actual size of the Au NPs (13 nm), and using simple mathematics it is possible to estimate the Au NPs surface coverage, being it about 2.9 × 10^11^ NPs/cm^2^. When considering the area of a single Au NP having a diameter of 13 nm, the estimated surface coverage is about 38%. This value, although being affected by a considerable error due to the simple calculations that for example do not take into account NPs size dispersity and crystalline structure, is quite close to the surface coverage value that was evaluated from a bare Au NPs layer deposited using the same experimental parameters (34%). So, the surface coverage of Au NPs is thereby qualitatively confirmed. 

**Figure 4:**
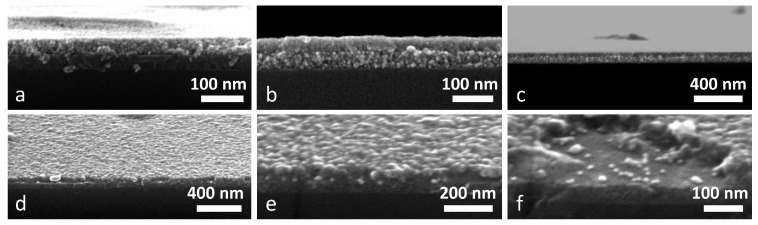
(**a**–**c**) SEM images of Au NPs layers embedded between TiO_2_ and NiOfilms: TiO_2_ is the bottom layer in (**a**) and the top layer in (**b**,**c**); (**d**–**f**) SEM images of Au NPs layers embedded between TiO_2_ (bottom) and ZnO (top) at different magnifications. Brighter spots correspond to Au NPs.

SEM characterization has been carried out performing the measurements in top view and in cross section, in order to evaluate the presence of the two layers, their thickness and morphology, and to examine the Au NPs distribution across the samples; all of the results are reported in Figure 4. Figure 4a–c shows the Au NPs layer embedded between TiO_2_ and NiO films: anatase film is the bottom layer in Figure 4a, and the top layer in Figure 4b,c. The morphology of the two oxides is clearly different: TiO_2_ films are more compact and smooth, while NiO layers have a more structured morphology, with the crystalline grains being clearly identifiable. Moreover, from the morphological difference between the two oxides, it seems that the NiO film has a higher porosity when compared to the TiO_2_ layer, and effectively this has been confirmed by the ellipsometric evaluation discussed before. The thicknesses evaluated from the SEM images is in good agreement with the predicted values: In Figure 4a, TiO_2_ and NiO films have been measured to be around 45 nm and 47 nm thick, respectively, while in Figure 4b, samples the evaluated thickness is 43 nm and 46 nm, respectively. Therefore, the target thickness of ~45 nm is confirmed. Au NPs can be seen as brighter spots, but since the difference in contrast with NiO crystals is quite low, it is sometimes difficult to distinguish them. Nevertheless, especially in Figure 4b, few bright particles in between the two oxide films can be recognized. From the low magnification image (Figure 4c), the high quality of the sandwich structure over few microns can be appreciated, and also Au NPs as brighter spots can be seen throughout the whole image, in between the two oxides.

Figure 4d–f show some images of a sample composed of Au NPs that are embedded between a bottom TiO_2_ layer and a top ZnO layer. Again, the difference in morphology between the two oxides can be seen, being ZnO rougher and TiO_2_ smoother, but also, Au NPs can be clearly seen due to the higher contrast difference. Bright circular spots exactly at the ZnO-TiO_2_ interface are seen in all three images, and their size has been estimated in the 10 nm–15 nm range, as consistent with the value of the as-synthesized particles. We previously reported that Au NPs deposited on glass tend to sinter upon thermal treatment, but when the Au NPs are covered with a metal oxide film, this provides a physical barrier that strongly reduces the temperature-driven sintering [10]. Figure 4f shows a picture of the double layer where a portion of the ZnO layer is missing (probably as a consequence of the sample cutting and handling): few Au NPs that are deposited over the TiO_2_ film can be easily seen, giving another proof of the metal NPs presence at the interface between the two oxides. Moreover, the thickness of TiO_2_ and ZnO layers has been evaluated as well, being 46 nm and 51 nm, respectively; again, the predicted thickness is hereby confirmed, even if the ZnO film is slightly thicker than expected, possibly because its high surface roughness makes the precise evaluation of the thickness quite challenging.

**Figure 5:**
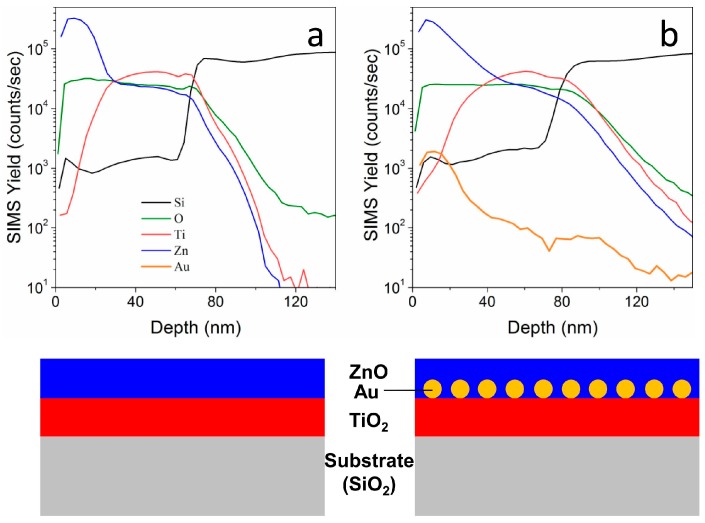
Secondary Ion Mass Spectrometry (SIMS) data showing depth profiles of different ions for (**a**) TiO_2_-ZnO and (**b**) TiO_2_-Au-ZnO thin films. A schematic representation of the respective sample structure is shown at the bottom.

SIMS provides another confirmation of the actual structure of the layered films (Figure 5): we evaluated the compositional depth profiles for two TiO_2_-ZnO films that were deposited on silicon substrates, with (b) and without (a) an Au NPs layer that is embedded in between. The total samples thickness has been estimated to be around 80 nm (based on Si and O signals), in good agreement with previous characterizations. The Zn signal is detected in both samples at the surface, while the Ti signal is centered around 40–60 nm far from the surface. The sample containing Au NPs (Figure 5b) shows Au signal that is centered about 20 nm from the surface, not exactly in between the two oxides, but slightly closer to the surface. This is understandable when considering the structure and morphology of the sample (Figure 4d–f), where it can be seen that Au NPs are laying on top of the anatase layer, and they are surrounded and submerged by the top ZnO coating. The schematic presented in Figure 5c shows the sample structure, highlighting the position of Au NPs. Moreover, with such thin layered structures the thickness estimation using SIMS is affected by a substantial error. However, a further confirmation of the results that were presented in the previous characterizations has been obtained.

We already studied the optical gas sensing properties of NiO, ZnO, and TiO_2_ film containing Au NPs [23,24], showing how the interaction of the target gas with the metal oxide matrix can be monitored by looking at the SPR of the Au NPs. Here, we embedded the Au NPs between two different metal oxide layers for studying the effect, if any, of their coupling on the optical gas sensing properties. For this preliminary study, H_2_ and CO have been tested as target gas because they were also used in our previous study on single metal oxides.

Among the different synthetized multilayer structures, the NiO-Au-TiO_2_ (NAT) and TiO_2_-Au-NiO (TAN) multilayers have been selected for the gas sensing measurements, because in our previous studies, the NiO-Au and TiO_2_-Au films showed good optical gas sensing properties toward H_2_ and CO [23,24].

**Figure 6:**
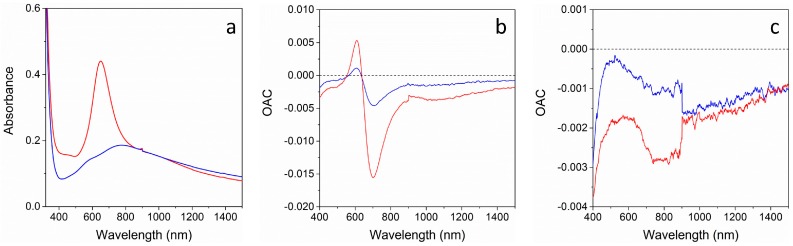
(**a**) Absorbance spectra of NiO-Au-TiO_2_ (NAT) (red line) and TiO_2_-Au-NiO (TAN) (blue line). Optical Absorbance Change (OAC) curves for (**b**) NAT sample and (**c**) TAN sample after exposure to 1% CO (blue lines) and 1% H_2_ (red lines) at 300 °C OT.

Figure 6 shows the absorption spectra of the two multi-layer structures and their Optical Absorbance Change (OAC) parameter, defined as the difference between absorbance during gas exposure and absorbance in air (OAC = Abs_Gas_ − Abs_Air_). The two samples respond rather differently: outside the 600–900 nm range, a decrease in absorption when exposed to both gases is seen for both samples, because of the interaction of the reducing gas with the NiO film [23]. Inside the 600–900 nm range, the NAT sample shows a sharp and strongly wavelength dependent signal, which is more intense for H_2_ when compared to CO, while for the TAN sample, only a weak modulation of the OAC curve is observed. This large difference can be related to the optical absorption spectra of the two samples (Figure 6a): NAT sample shows a narrow and sharp Au SPR peak, possibly due to a partial detachment of Au NPs when depositing the top TiO_2_ layer, while TAN optical spectrum presents a much broader, weaker, and red shifted plasmon peak. As a consequence, the difference from the spectra collected during gas exposure and the spectra collected in air, i.e., the OAC parameter, is strongly affected, being much higher when the optical spectrum has steep features, and much lower when the optical spectrum has plainer features.

In any case, for both of the samples, a reversible response for both gases is observed and some distinctive wavelengths corresponding to maximum and minimum (or null) response can be identified, theoretically permitting selective gas recognition through an appropriate choice of the analysis wavelength [25,26].

**Figure 7:**
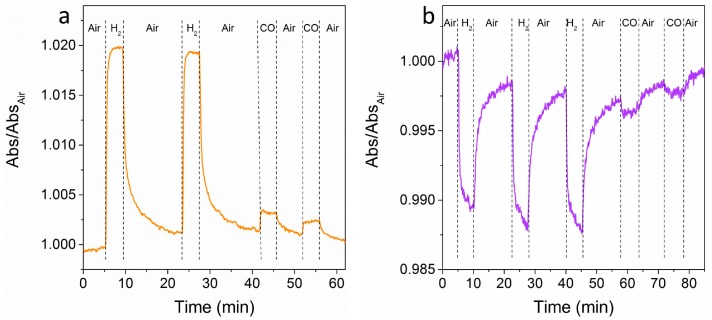
Dynamic tests after several air-gas-air cycles for: (**a**) NAT sample at 609 nm; (**b**) TAN sample at 580 nm.

Figure 7 shows time-resolved tests at a fixed wavelength for multiple air-gas-air cycles. The wavelengths have been selected for obtaining a very high signal for H_2_ and the smallest signal for CO, in order to demonstrate the selectivity of the sensor. An easily detectable signal for both of the gases is observed, which is much higher for hydrogen when compared to CO, as predicted from OAC curves (see Figure 6b,c), with relatively fast response times (between 30 s and 60 s) and acceptable recovery times (between 60 s and 90 s).

## 4. Conclusions

Gold NPs have been successfully embedded at the interface between two different semiconducting oxides with an easy and straightforward procedure: first, monodisperse Au nanocrystals are synthesized with standard colloidal techniques, purified, and then deposited over a pre-functionalized sol-gel based metal oxide thin film; eventually, this structure is covered with a second oxide layer. The surface coverage of Au colloids can be easily tuned, and optical spectroscopy measurements that are coupled to morphological characterizations confirm the successful embedding of the metal spheres in between the two oxides, with the predicted surface coverage. In this nano-architecture, Au NPs are facing two different materials, with possible new interesting properties due to the multiple noble metal/metal oxide interfaces. These multilayered structures represent a high level of materials engineering, providing accurate control on NPs morphology, organization, and proper interface with the desired semiconducting material. Moreover, this process can be easily extended to a great variety of multilayered structures, which can find applications in several fields, including optical sensors, catalysts, and optoelectronic devices in general.
